# The Role of Dimethyl Sulfoxide (DMSO) in Gene Expression Modulation and Glycosaminoglycan Metabolism in Lysosomal Storage Disorders on an Example of Mucopolysaccharidosis

**DOI:** 10.3390/ijms20020304

**Published:** 2019-01-14

**Authors:** Marta Moskot, Joanna Jakóbkiewicz-Banecka, Anna Kloska, Ewa Piotrowska, Magdalena Narajczyk, Magdalena Gabig-Cimińska

**Affiliations:** 1 Institute of Biochemistry and Biophysics, Polish Academy of Sciences, Laboratory of Molecular Biology, Wita Stwosza 59, 80-308 Gdańsk, Poland; m.gabig@biol.ug.edu.pl; 2 Department of Medical Biology and Genetics, Faculty of Biology, University of Gdańsk, Wita Stwosza 59, 80-308 Gdańsk, Poland; joanna.jakobkiewicz-banecka@biol.ug.edu.pl (J.J.-B.); anna.kloska@biol.ug.edu.pl (A.K.); 3 Department of Molecular Biology, Faculty of Biology, University of Gdańsk, Wita Stwosza 59, 80-308 Gdańsk, Poland; ewa.piotrowska@biol.ug.edu.pl; 4 Laboratory of Electron Microscopy, Faculty of Biology, University of Gdańsk, Wita Stwosza 59, 80-308 Gdańsk, Poland; magdalena.narajczyk@biol.ug.edu.pl

**Keywords:** lysosomal storage diseases, mucopolysaccharidosis type III (MPS III, Sanfilippo syndrome), dimethyl sulfoxide (DMSO), glycosamoninoglycans (GAGs) therapy

## Abstract

Obstacles to effective therapies for mucopolysaccharidoses (MPSs) determine the need for continuous studies in order to enhance therapeutic strategies. Dimethyl sulfoxide (DMSO) is frequently utilised as a solvent in biological studies, and as a vehicle for drug therapy and the in vivo administration of water-insoluble substances. In the light of the uncertainty on the mechanisms of DMSO impact on metabolism of glycosaminoglycans (GAGs) pathologically accumulated in MPSs, in this work, we made an attempt to investigate and resolve the question of the nature of GAG level modulation by DMSO, the isoflavone genistein solvent employed previously by our group in MPS treatment. In this work, we first found the cytotoxic effect of DMSO on human fibroblasts at concentrations above 3%. Also, our results displayed the potential role of DMSO in the regulation of biological processes at the transcriptional level, then demonstrated a moderate impact of the solvent on GAG synthesis. Interestingly, alterations of lysosomal ultrastructure upon DMSO treatment were visible. As there is growing evidence in the literature that DMSO can affect cellular pathways leading to numerous changes, it is important to expand our knowledge concerning this issue.

## 1. Introduction

Lysosomal storage diseases (LSDs) are a group of over 50 rare inherited metabolic disorders that appear from defects in lysosomal function, leading usually to deficiency of a single enzyme required for the metabolism of lipids, glycoproteins or glycosaminoglycans (GAGs) [1,2]. Alterations in the metabolism of GAGs due to mutations in genes coding for enzymes involved in degradation of these compounds cause severe inherited metabolic diseases known as mucopolysaccharidoses (MPSs). Impaired hydrolysis of GAGs leads to their accumulation in cells of patients, which results in a progressive damage of the affected tissues and organs, including the heart, respiratory system, bones, joints and central nervous system (CNS) [3]. Remarkably, studies of the neurodegeneration process in MPSs have shown increased levels of protein markers of Alzheimer’s disease and other tauopathies in brain areas involved in learning and memory (primarily dentate gyrus and medial entorhinal cortex) of MPS IIIA, MPS IIIB [4] and MPS IIIC mice [5].

Bone marrow or haematopoietic stem cell transplantations and enzyme replacement therapy (ERT) are the only approved treatments for MPS [6]. However, neurological symptoms, developing due to GAG accumulation in CNS, cannot be managed by ERT owing to an inefficient delivery of proteins through the blood–brain barrier (BBB) [7]. Among a few different therapeutic strategies, for example, gene therapy or direct delivery of the enzyme in the cerebrospinal fluid, some other approaches can be distinguished [8]. To this end, a small inhibitory molecule prone to penetrating the BBB is employed. Together with the presence of about 3% of the residual activity of particular hydrolase, this proposal allows restoration of the balance in the metabolism of macromolecules, which leads to the lack of stored material within lysosomes. Implementation of this strategy is called substrate reduction therapy (SRT) [9]. The efficiency of this rationale has been observed using the flavonoid genistein (5,7-dihydroxy-3-(4-hydroxyphenyl)-4*H*-1-benzopyran-4-one) in Sanfilippo disease, and miglustat (Zavesca^®^) in Gaucher disease [10]. Many investigations have revealed that due to pleiotropic effects, genistein can be used to modulate pivotal mechanisms in human cells, for instance, the cell cycle [11], metabolism of macromolecules [12] or biogenesis and activity of lysosomes [13,14]. Because patients suffering from different types of MPSs have many defects in cellular processes as mentioned above, this isoflavone is considered as a novel therapeutic agent, especially in a combination therapy with an enzyme.

One of the important aspects of our research work so far, in which we tested genistein for implementation in MPS treatment, was to use a solvent appropriate for this substance, which is at the same time well tolerated by the human body during consumption. Dimethyl sulfoxide (DMSO), a by-product of the wood industry, has been used as a commercial solvent since 1953, while the history of DMSO as a pharmaceutical began in 1961 [15,16]. From this time, it has been frequently utilised as a diluent in biological studies and as a vehicle for drug therapy and in the in vivo administration of water-insoluble substances. DMSO has been used successfully in many human therapeutic situations, such as in the treatment of interstitial cystitis, dermatological, urinary, pulmonary, rheumatic and renal manifestations, amyloidosis, gastrointestinal diseases, musculoskeletal disorders and rheumatologic and dermatologic diseases, as a topical analgesic, and others [17,18]. Importantly, DMSO crosses the BBB [19]; thus, it has been effective in the treatment of traumatic brain oedema [20]. This feature is not without significance even when it comes to using it as a solvent for substances that are to cross the BBB, such as genistein utilised in the treatment of LSDs with neurological manifestation. DMSO is known as an agent affecting GAG synthesis and excretion [21,22,23], and changes in the biochemical composition of DMSO-treated cells with regard to sulfated and nonsulfated GAGs have been observed [24]. On the other hand, the side effects of DMSO, apparent from its use in the laboratory (both in vitro and in vivo) and in clinical settings, are reported [18], though its mechanisms of action have not yet been fully elucidated. These are often simply neglected; therefore, when working with DMSO, one might be aware of the experience of other groups who are using it, even in very varied contexts.

The first reports indicated inhibition of GAG synthesis by DMSO in Chinese hamster ovary (CHO) cells [24] and in HLH60/HGPRT leukaemia cells. According to Constantopoulos et al., DMSO inhibits cell proliferation, but also GAG synthesis and secretion in cultured glioma [25] and this effect was comparable to results observed in rat prostate adenocarcinoma [22]. Surprisingly, in the same study, adenocarcinoma cells that acquired the capacity to grow in higher DMSO concentration secreted excessive amounts of glycosaminoglycans.

In the light of the above-described facts and uncertainty on the mechanisms of DMSO regulation of GAG metabolism, in this work we have tried to resolve the question of the nature of GAG level modulation by DMSO, the genistein solvent employed by our group in MPS treatment.

## 2. Results

### 2.1. Cytotoxicity of DMSO in In Vitro Cell Cultures

DMSO is usually well tolerated with no observable toxic effects to cells at 0.1% final concentration and is widely used as a solvent for various pharmacological agents at concentrations of 0.05–1.5%. Thus, to test the influence of DMSO on human dermal fibroblasts, HDFa and MPS IIIA and IIIB, viability and proliferation, we selected concentrations from 0.01% to 5% *v*/*v*.

**Figure 1:**
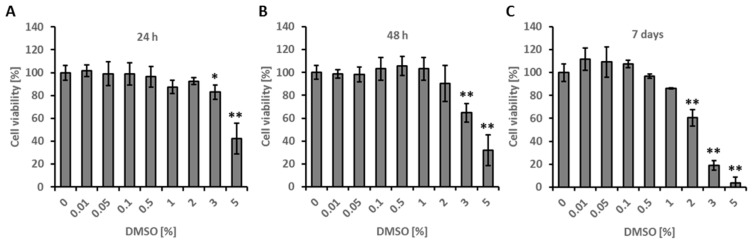
Cell viability following exposure to DMSO. Cells were grown in medium supplemented with increasing DMSO concentrations for 24 h (**A**), 48 h (**B**) or 7 days (**C**). Cell viability was assessed in MTT assay and was calculated as relative to control cells grown in culture medium without DMSO. Values represent means of at least two independent experiments (each run in triplicate) with bars representing standard deviation. Statistical significance was tested by one-way ANOVA followed by Tukey’s multiple comparison test; * *p* < 0.05 and ** *p* < 0.01 vs. control cells without DMSO treatment.

After 24 h of culture, DMSO concentrations of 3% *v*/*v* had little cytotoxic effect on tested cells, and inhibited growth to 81%, compared to untreated control culture (Figure 1). Exposure of cultures for 48 h to concentrations reaching 1% *v*/*v* DMSO had no significant effect, and cell viability was even increased, as revealed by MTT (3-[4,5-dimethylthiazol-2-yl]-2,5 diphenyl tetrazolium bromide) assay. DMSO at 3% and 5% *v*/*v* concentration reduced cell growth in both times of exposure to 81% and 55%, and 60% and 44%, respectively.

After seven days, fibroblast proliferation was reduced 85% and 57% in case of 1% and 2% *v*/*v* DMSO, while 3% *v*/*v* resulted in 16% of viability and 5% concentration was lethal for the whole culture. DMSO at 0.01%, 0.05%, 0.1% and 0.5% *v*/*v* had no impact on cell growth and proliferation.

### 2.2. Fibroblast Gene Expression Profiling upon DMSO Treatment

**Figure 2:**
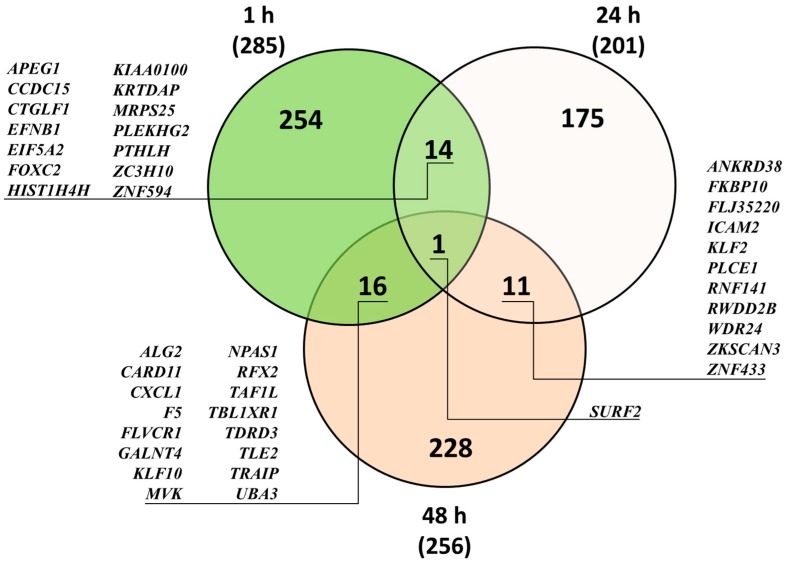
Venn diagram of HDFa whole genome modulated transcripts upon DMSO treatment. Expression level was assessed by microarray analysis. Numbers in parentheses represent the amount of DEGs (FC ≤ 0.7 and FC ≥ 1.3) for each time point, *n* = 3. Numbers in overlapping parts of diagram represent shared genes (listed).

Microarray studies of the impact of DMSO on HDFa transcriptome with Illumina’s Human HT-12 v4 Expression BeadChips were performed for DMSO concentration, 0.05% *v*/*v*, most frequently applied when used as a solvent. This assumption was based on medium impact of DMSO at higher concentrations on processes like GAG synthesis. Gene expression analysis revealed 285, 201 and 256 differentially expressed genes (DEGs) upon 1, 24 and 48 h exposure, respectively, to 0.05% *v*/*v* DMSO, compared to untreated control (Table 1). Within these genes, more than 87% had upregulated expression after 1 and 24 h treatment: 249 and 176, respectively. In the case of 48 h exposure to DMSO, the effect was more balanced, reaching ca. 58% and 42% for up- and downregulated genes, 148 and 108, respectively. Moreover, we found a few genes with expression modulated after more than one time period (Figure 2), with *SURF2* expression changed under all conditions. Typically, for the whole analysis, modulation of gene expression was rather moderate, with minimum and maximum changes around 0.5- and 2-fold, respectively (Table 2), but it was difficult to define common biological processes for the products of genes with mostly altered expression.

There were also only a few genes differentially expressed after DMSO treatment whose products are involved in lysosomal, glycosaminoglycan or sphingolipid metabolism pathways (Table 3). Denoted modulation in expression level was around 0.6–0.7 and 1.3–1.7 for down- and upregulation, respectively. The products of positively regulated transcripts might be mainly classified under glycan biosynthesis and metabolism, glycosphingolipid biosynthesis, sphingolipid metabolism and sphingosine biosynthesis, while the products of negatively regulated transcripts might be involved in lysosomal metabolism, with special focus on *ZKSCAN3*, a master repressor of autophagy.

DMSO is frequently used in liver cell cultivation and is thought to affect the expression of various cytochrome P450 (CYP450) isoforms by inducing or preserving cellular differentiation. In the course of our analysis, no changes were seen in the expression level of genes coding for CYP450, which were observed in cases of human, rat and murine hepatocytes. But as has been shown, human dermal fibroblasts express low levels of *CYP* [26].

### 2.3. Gene Ontology (GO) Analysis of DMSO-Modulated Transcripts

Results of DEGs analysis with AmiGO [27] and the Panther Classification System [28] ranked the list of GO terms with percentage of gene hits against total number of analysed genes with modulated expression. In all tested conditions, GO annotations for groups of differentially expressed genes were mostly repetitive (Table 1), both in the case of same type modulation but also between up- and downregulated gene sets.

Molecular function was mostly represented by binding (GO:0005488) and catalytic activity (GO:0003824): 19–29% and 14–23% for downregulated genes, respectively. At approximately similar levels, those annotations were enriched for upregulated genes, reaching 22–31% and 22–28% for binding and catalytic activity, respectively. Transporter activity (GO:0005215) and signal transducer activity (GO:0004871) were also common for all up- and downregulated gene sets, besides fold change (FC) of ≤0.7 for 24 h incubation with 0.05% DMSO, but with a minor contribution (1.3–7.8%).

Biological processes were predominantly exhibited by cellular process (GO:0009987) and metabolic process (GO:0008152), ranging from 38–48% and 33–34% for upregulated genes, and 32–44% and 29–44% for negatively-regulated genes, respectively. Other shared processes were biological regulation (GO:0065007), cellular component organisation or biogenesis (GO:0071840), response to stimulus (GO:0050896), developmental process (GO:0032502), localisation (GO:0051179) and multicellular organismal process (GO:0032501), with gene hit against total number of genes around 10%, (precisely 3–19%) for all analysed gene sets.

Cellular component analysis revealed cell part (GO:0044464) and organelle (GO:0043226) to be mostly represented between all gene sets: 28–36% and 18–27% for up- and 19–28% and 18–20% for downregulated genes, respectively. Also in this shared group was macromolecular complex (GO:0032991) with 7–14% and 3–14% gene hits, for up- and downregulated genes, respectively. For positively modulated transcripts in common were also extracellular region (GO:0005576) and extracellular matrix (GO:0031012) with 1–4% enrichment.

Major distinguishable protein classes coded by modulated genes were transcription factor (PC00218), nucleic acid binding (PC00171), hydrolase (PC00121) and transferase (PC00220), representing between 7% and 15% of whole transcripts. Upregulated genes were also classified as enzyme modulator (PC00095), signalling molecule (PC00207), receptor (PC00197), cytoskeletal protein (PC00085), transporter (PC00227), extracellular matrix protein (PC00102), cell adhesion molecule (PC00069), as were downregulated genes from samples treated with DMSO for 48 h. All these groups were enriched by 1–7%. Worthy of mention is the high number of genes assigned as transcription factor, signalling molecule and receptor, which may have special impacts on cell metabolism.

Although GO analysis indicated many pathways related to genes with modulated activity, especially for positive modulation (44 pathways for 1 h treatment with DMSO, 60 for 24 h and 35 for 48 h, with 249, 176 and 148 upregulated genes, respectively, for particular conditions), each pathway was represented by only 1–3 genes. Only a limited number of pathways were enriched with more than five genes, as in analysis of transcripts with upregulated expression upon 1 h and 24 h DMSO treatment. While the percentage of gene hits against total number of genes did not exceed 5% (with only one exception: 9.5% for downregulated gene set analysis after 24 h DMSO incubation), no prevalent pathways were distinguished.

Gene Set Enrichment Analysis [29] did not reveal any gene sets significantly enriched at FDR < 25%. One of the reasons may be a small number of DEGs.

### 2.4. Kinetics of GAG Synthesis upon DMSO Treatment in MPS and HDFa Fibroblasts

**Figure 3:**
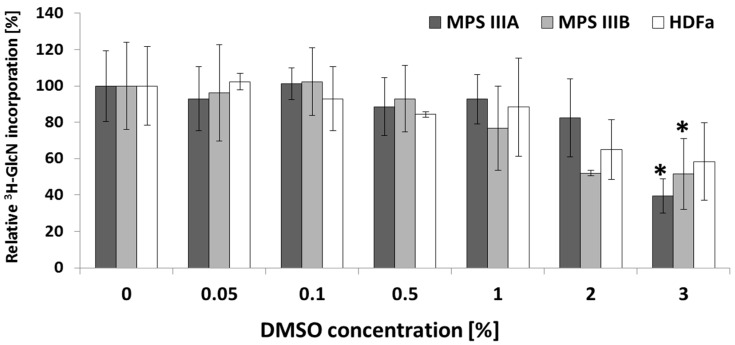
Kinetics of GAG synthesis upon DMSO treatment. Relative level of glycosaminoglycan synthesis in HDFa, MPS IIIA and fibroblasts after 72 h treatment with different concentrations of DMSO measured by incorporation of [3H]GlcN. Values represent means of at least two independent experiments (each run in triplicate) with bars representing standard deviation. Statistical significance was tested by Kruskal–Wallis test followed by Dunn–Bonferroni post hoc; * *p* < 0.05 and vs. control cells without DMSO treatment.

No significant differences in GAG synthesis level (10–20%) have been observed upon treatment with low DMSO concentrations; however, higher concentrations, 2% and 3%, resulted in 20–60% reduction in GAG synthesis, depending on the cell line tested (Figure 3). The most substantial reduction, 61%, was noted for MPS IIIA fibroblasts upon treatment with 3% DMSO. A statistically significant decrease in glucosamine, D-[1-3*H*] incorporation was achieved only for MPS IIIA and B fibroblasts cultivated with 3% DMSO (Kruskal–Wallis test followed by Dunn–Bonferroni post hoc; * *p* < 0.05) and versus control cells without DMSO treatment.

### 2.5. Electron Microscopy Studies of Lysosomal Compartment

**Figure 4:**
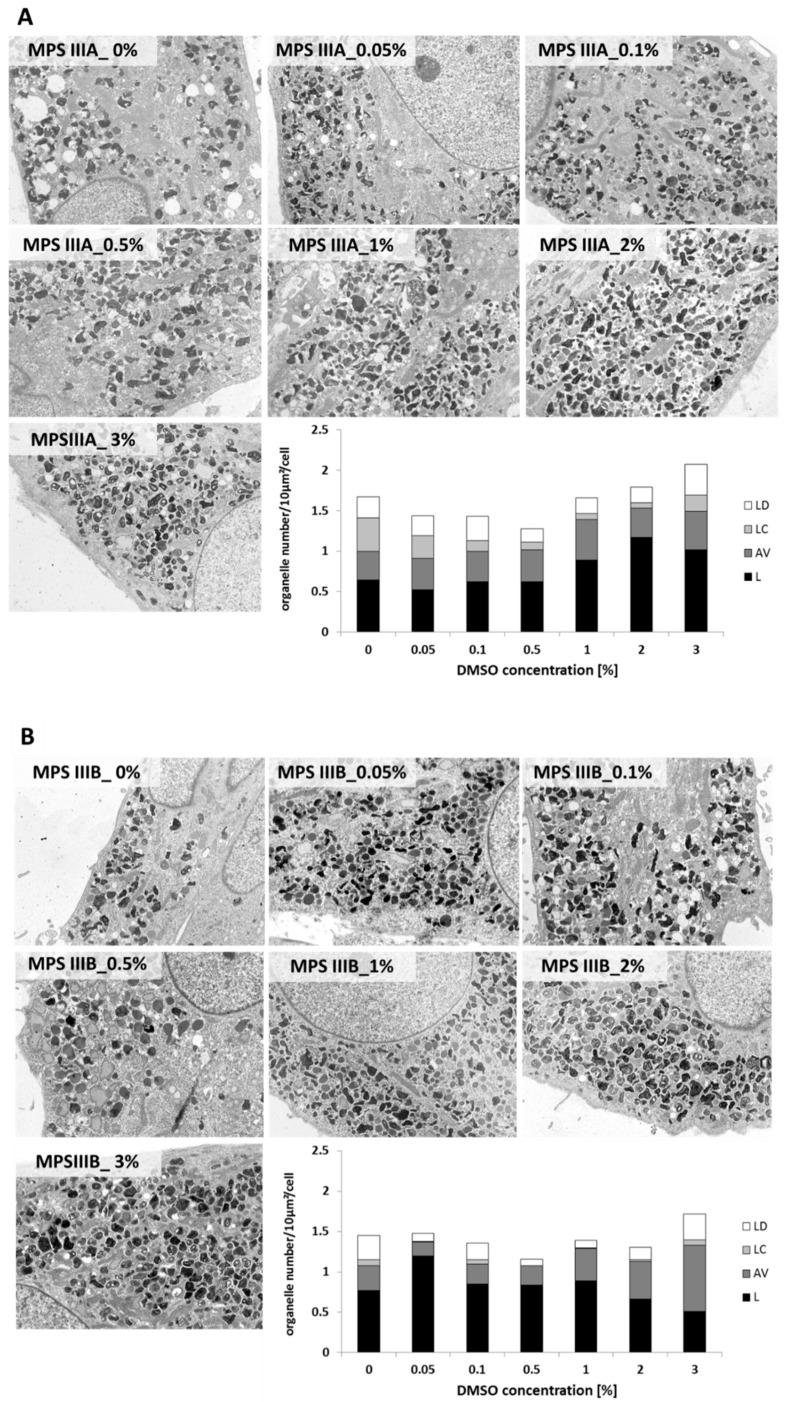

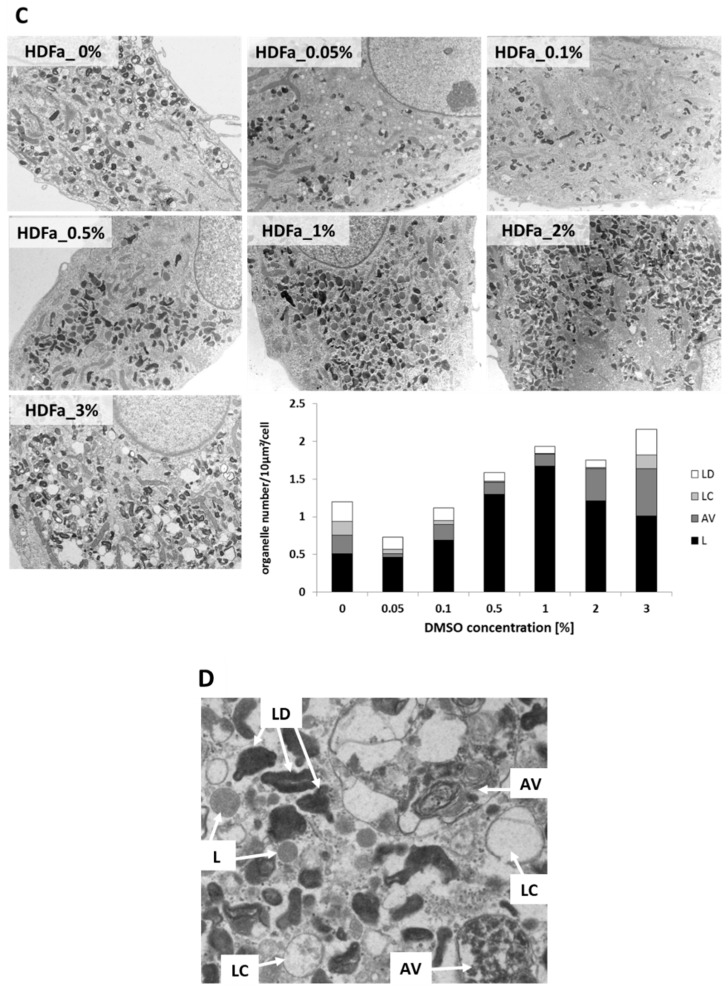
Ultrastructure of MPS III A, B and control (HDFa) fibroblasts (Panels (**A**–**C**), respectively). Micrographs in TEM after treatment with various DMSO concentrations for 48 h. Images were collected from at least 20 cells with magnification 1650×. Typical lysosomal structures are presented in panel (**D**). L—lysosome, AV—autophagosomal vacuoles, LC—carbohydrate-storing lysosomes, LD—lysosomes of amorphous flocculent and electron-dense structure.

Transmission electron microscopy (TEM) of MPS patient and control fibroblasts enabled visualisation of lysosomes (L), carbohydrate storing lysosomes (LC), lysosomes of amorphous flocculent and electron dense structure (LD) and autophagosomal vacuoles (AV). Mean numbers of lysosomal structures per 100 μm^2^ cell cross-section are presented (Figure 4). Organelles of special interest were LC, due to potential GAG storage.

The most notable changes in fibroblast ultrastructure were seen at the highest DMSO concentration tested (3%); however, this treatment also resulted in visible alterations in cell morphology. The most prevalent organelles denoted were single lysosomes. A concentration-dependent increase in the number of these structures per cell was observed in MPS IIIA (panel A), and even more in HDFa cell cultures (panel C). In contrast, a subtle decrease was seen in MPS IIIB fibroblasts (besides almost 50% increase for 0.05% *v*/*v* DMSO compared to untreated control) (panel B). The accumulation of structures morphologically defined as carbohydrate-storing lysosomes was rather minor even in MPS cells, and any changes in this vesicle number were difficult to define. The number of lysosomes that accumulated electron-dense structure was slightly concentration dependently decreased in MPS IIIB and HDFa fibroblast, while in MPS IIIA, it was constant. In the case of AVs, we observed an increase in this fraction for MPS IIIB and HDFa fibroblasts, with no differences in MPS IIIA cells. TEM analysis did not reveal any significant differences in the size of lysosomal structures, other than LCs in 3% DMSO-treated HDFa, which were visibly larger. Examples of lysosomal structures occurring in MPS cells are presented in Figure 4.

## 3. Discussion

Research on the implementation of the nonenzymatic SRT using GAG metabolism modulators to treat MPSs with neurological symptoms is one of the investigation concepts initiated with various flavonoids [7,8]. We demonstrated previously that natural flavonoids, such as genistein (5,7-dihydroxy-3-(4-hydroxyphenyl)-4*H*-1-benzopyran-4-on), kaempferol (3,5,7-trihydroxy-2-(4-hydroxyphenyl) chromen-4-one) and daidzein (7-hydroxy-3-(4-hydroxyphenyl) chromen-4-one) significantly inhibit synthesis and reduce the level of GAGs in cultures of HDFa cells and fibroblasts of MPS patients and mice [7,8,30]. SRT could be effective in the management of CNS-related symptoms of MPS, especially if a small molecule, capable of crossing the BBB, is employed.

Since DMSO has been widely used as a vehicle for the delivery of various molecules in cultured cells, in vivo experiments and in medicine (particularly for conditions such as scleroderma, and rheumatoid- and osteoarthritis), it is worth verifying more deeply its potential impact on biological processes such as glycosaminoglycan biosynthesis.

### 3.1. Cytotoxic Effect of DMSO on Human Fibroblasts at Higher Concentrations

In the course of our studies, there was no cytotoxic effect of DMSO in concentrations similar to those commonly assumed as safe and routinely used in cell cultures [31]. DMSO concentrations up to 3% *v*/*v* were well tolerated by HDFa. Similar results were obtained in goat skin fibroblasts, where cells survived beyond 3% DMSO, but concentrations regarded as higher (0.5–3% *v*/*v*) led to reductions in cell viability in a dose-dependent manner. In many studies, 0.1% *v*/*v* DMSO in growth media was established as the safest, while DMSO concentrations more than 1% *v*/*v* have been shown to be toxic for most mammalian cell types when used in in vitro culture assays. Even so, certain studies have shown that lower concentrations of DMSO (0.05–0.2% *v*/*v*) may enhance proliferation in some cell types, such as ovarian cancer cells [32]. In fibroblasts obtained from Niemann–Pick patients, cellular division was blocked and DMSO above 2% *v*/*v* was cytotoxic [33].

### 3.2. Potential Role of DMSO in Regulation of Biological Processes on Transcriptional Level

DMSO is generally considered to be genetically inactive and is thus very frequently used as a solvent in drug-screening assays. In concentrations of 1.5–5% *v*/*v*, or even 10% *v*/*v*, it is frequently applied to induce differentiation of various cell lines [34], including fibroblasts [31,32]. Differentiation of fibroblasts into myofibroblasts is one of the key factors for wound healing and is accompanied by release of several types of cytokine [33]. The results of Morley and Whitfield suggest that DMSO-induced Ca^2+^ release from intracellular stores may play a role in the induction of cell differentiation in primary cultures and in cells of a variety of established lines [35]. However, the ability of DMSO to induce cell differentiation indicated that this compound might exert an influence at the genetic regulation level; by gene ontology analysis conducted in the course of our studies, we did not find any pathways or biological processes connected to cell differentiation (Table 1). Gene expression analysis for sequences with most modulated expression did not derive any specified common process or cellular pathway. It must be emphasized that for 0.05% *v*/*v* DMSO, changes in transcriptional level were rather subtle, with fold change value 0.6–0.7 and 1.3–1.7 for down- and upregulation, respectively (Table 2 and Table 3). Importantly, both microarray and real-time qRT-PCR analyses of GAG metabolism- and lysosome-associated genes gave similar results, indicating accuracy of both methods (Table 3). Also, percentage of modulated transcripts was minor and ranged less than 1% of well-defined sequences. The only gene that appeared to be regulated at all three time points was *SURF2*, a housekeeping gene, but expression modulation was rather subtle.

Among the biological processes enriched in the present analysis, the predominant were the metabolic process (GO:0008152) and cellular process (GO:0009987), where nearly 40% of modulated transcripts were involved. Nevertheless, the impact on the biological regulation (GO:0065007) process might be crucial, with 5.9–16.2% genes showing altered expression, and also protein class GO terms, such as transcription factor (PC00218) and nucleic acid binding (PC00171), showing 5–14.7% and 6.9–13.9% of modulated transcripts, respectively. For molecular function, the most enriched terms were binding (GO:00054888) and catalytic activity (GO:0003824), where 19–31.1% and 14.3–27.7% of genes with altered expression were involved, respectively. All these results display the potential role of DMSO in regulation of biological processes at the transcriptional level as a transcription factor modulator.

### 3.3. Moderate DMSO Impact on GAG Synthesis

One open question remained the influence of DMSO on the synthesis of glycosaminoglycans. The results of our research performed on fibroblasts provided by MPS patients and on healthy controls displayed GAG synthesis reduction, but only for higher (above 1% *v*/*v*) DMSO concentrations. The obtained data were not statistically significant. Furthermore, gene expression analysis followed by GO revealed only few DEGs involved in GAG metabolism what might be due to applied concentration of DMSO, 0.05%, that did not result in GAG synthesis reduction. No significant enrichments in cellular components or biological processes, such as lysosome or lysosomal biogenesis, besides *ZKSCAN3*, an inhibitor of autophagy, were noted, which might indicate no impact of DMSO on GAG degradation at this concentration, either. All these data demonstrate low impact of DMSO on GAG synthesis modulation at concentrations employed when it is used as a solvent for biological applications.

Treatment of rat prostate adenocarcinoma PAIII cells with 1.5% DMSO decreased both the secretion of GAGs into the medium and also the incorporation of [3H]glucosamine into the cellular GAG level. However, in cells adapted to higher DMSO concentrations (2.5% *v*/*v*), PAIII-DMSO, the quantity of GAGs secreted into the medium was increased to approximately twice the level of untreated PAIII cells. In unadapted PAIII cells, exposure to 2.5% *v*/*v* DMSO reduced the amount of GAGs shedding into the culture medium to 18% of control values [22]. Those differences presented between rat prostate adenocarcinoma cells resistant to high DMSO concentrations and the parental line suggest selection of a genetically diverse subpopulation or induction of a compensation mechanism by constant exposure to DMSO. While in the course of our studies modulation of GAG synthesis was estimated only on the basis of cellular content, some difficulties may arise in analysis of the examined solvent effect. Here, the cellular level of synthesised GAGs was decreased.

The effect of DMSO was studied also on another lysosomal storage disorder, Niemann–Pick disease (NPD) type B and C. Sphingomyelinase (SMase) activity in human skin fibroblasts from NPD patients was increased up to 230% of control by 2% *v*/*v* DMSO, while the cell growth was inhibited. Other lysosomal hydrolases displayed a rather inconsiderable increase in activity [36] and the effects of DMSO on sphingomyelinase activity in fibroblasts from Niemann–Pick type B patients were less than those of a type C. Comparable results were obtained from studies with NPD type B GM 3393 cell line and indicated that activities of lysosomal hydrolases, especially β-galactosidase, were found to be upregulated. However, observed changes in the levels of the activities of these enzymes after DMSO treatment were still lower than the DMSO-induced increase in SMase activity in cells from normal and type C patients [37].

### 3.4. Alterations of Lysosomal Ultrastructure upon DMSO Treatment

Previous electron microscopy reports on fibroblasts from MPS patients revealed the accumulation of enlarged vacuolar structures containing glycosaminoglycans and lipids forming characteristic multiconcentric lamellae. Analysis was performed in the course of this study of both MPS and HDFa with TEM-enabled visualisation of representative lysosomal structures. It is worth noting that we observed a concentration-dependent decrease in the number of carbohydrate-storing electron lucent lysosomes (LCs) mostly in MPS IIIBa and HDFa, but also in MPSIIIA fibroblasts after six-day exposure to DMSO, with highest reductions in 0.5% and 1% DMSO concentrations relative to untreated controls (Figure 4). In MPS IIIB and HDFa cells, treatment with those concentrations resulted in an almost undetectable level of carbohydrate-storing lysosomes. This result might be crucial, as electrolucent LCs seem to be lysosomes loaded with GAGs. There was also noticeable increase in the number of lysosomes, indicating upregulated lysosomal metabolism, detected especially in HDFa, but also in MPS IIIA cells. MPS IIIB fibroblasts have shown considerably higher numbers of single lysosomes for 0.05% DMSO only, while in the case of MPS IIIA and HDFa, at this concentration, there were no differences from the untreated control. The decrease in the number of carbohydrate-storing lysosomes was comparable to that achieved in studies on the influence of selected flavonoids and their derivatives on GAG storage in MPS cell lines [7,27,38,39], where the potential therapeutic efficacy of natural compounds was tested.

## 4. Materials and Methods

### 4.1. Cell Lines, Culture Media, Supplements and Flavonoid Solutions

HDFa (Cascade Biologics, Portland, OR, USA) and MPS IIIA and IIIB fibroblasts (Children’s Memorial Health Institute, Warsaw, Poland) were cultured in Dulbecco’s modified Eagle’s medium (DMEM, Sigma-Aldrich, Steinheim, Germany) supplemented with 10% foetal bovine serum (FBS) and 1% antibiotic/antimycotic solution (Sigma-Aldrich, Steinheim, Germany), 5% CO_2_ at 37 °C. For experimental procedures, cells at passages were plated to a confluency of approximately 80% and grown at 37 °C in humid atmosphere containing 5% CO_2_.

### 4.2. Cytotoxicity and Proliferation Assay

MTT (3-[4,5-dimethylthiazol-2-yl]-2,5-diphenyltetrazolium bromide) assay was performed to estimate cell growth and proliferation. HDFa cells were seeded 6 × 10^3^ per well (in cytotoxicity assay) or 10^3^ cell per well (in proliferation assay) in flat-bottomed, 96-well plates and treated with 0.01%, 0.05%, 0.1%, 0.5%, 1% and 5% *v*/*v* DMSO as control for 24 h, 48 h, and 7 days at 37 °C. After the incubation period, the medium was replaced with RPMI (Sigma-Aldrich) supplemented with MTT (1 mg/mL) for another 4 h. The purple formazan crystals were dissolved in 150 µL DMSO, and absorbance was determined at 570 nm using a Wallac 1420 Multilabel Counter (Perkin Elmer, Waltham, MA, USA).

### 4.3. RNA Extraction

Total RNA was extracted using the High Pure RNA Isolation Kit (Roche Applied Science, Indianapolis, IN, USA) following the manufacturer’s instructions. The quality and quantity of each RNA sample was evaluated using the RNA 6000 Nano Assay on an Agilent 2100 Bioanalyser (Agilent Technologies Inc., Agilent Technologies, Loveland, CO, USA).

### 4.4. Microarray Assays for mRNA Analysis

Whole genome microarray analysis of three biological replicates (n) was performed using Illumina’s Human HT-12v3 Expression BeadChips, for more than 47,000 genes and sequences (Illumina Inc., San Diego, CA, USA) as reported in our previous publication [13]. 1 × 10^5^ HDFa cells were seeded and grown overnight and then treated with 0.05% DMSO for 1, 24 and 48 h, assigned formerly as vehicle control (K2), followed by RNA isolation. Cells harvested in medium nonsupplemented with DMSO were treated as control, formerly named pure control, K1. The assay performance, data extraction and statistical analysis were performed as previously described [13]. Genes with FC expression greater than or equal to 1.3 or lower than or equal to 0.7 were described as modulated. Gene Ontology analysis and data visualisation were performed on the upregulated and downregulated gene lists separately, using the web tools GOrilla (http://cbl-gorilla.cs.technion.ac.il/) restricting the output to biological process, molecular function and protein class. Additionally, AmiGO [27] GO analysis tool, together with Panther Classification System [28], were applied to verify biological process, molecular function, cellular component and pathways (Biocarta, KEGG, Reactome, GO_biological process, GO_cellular component) represented by groups of genes with modulated expression. Gene Set Enrichment Analysis (GSEA) was performed as previously described [29]. A nominal *p*-value < 0.01 and a false discovery rate (FDR) <0.25 were used to assess the significance of the enrichment scores. The microarray expression data used in this study had GEO accession numbers GSE34074 [13].

### 4.5. Real-Time qRT-PCR Assays for mRNA Analysis

Quantitative real-time Reverse Transcription PCR (real-time qRT-PCR) was performed to measure the mRNA levels of the genes coding for GAG and lysosomal metabolism. Total RNA was reverse-transcribed into cDNA using Transcriptor First Strand cDNA Synthesis Kit (Roche Applied Science, Indianapolis, IN, USA), according to the manufacturer’s instructions. Real-time qRT-PCR was carried out with TaqMan Gene Expression Assays (Thermo Fisher Scientific, Foster City, CA, USA) with catalog numbers as follows: #4426961 (Hs00904548_m1, *ALG2*), #4331182 (Hs00970128_m1, *ALG12*), #4331182 (Hs00406036_m1, *ARSK*), #4331182 (Hs00155245_m1, *B4GALT1*), #4331182 (Hs01921028_s1, *CHST2*), #4331182 (Hs00248144_m1, *CHST15*), #4331182 (Hs00609162_m1, *EXT1*), #4331182 (Hs00975732_m1, *GALNS*), #4331182 (Hs00189537_m1, *GALNT2*), #4331182 (Hs00559726_s1, *GALNT4*), #4331182 (Hs99999909_m1, *GALNT6*), #4331182 (Hs00226436_m1, *GALNT12*), #4448892 (Hs00166197_m1, *GM2A*), #4331182 (Hs00428900_m1, *MAN2C1*), #4331182 (Hs01100653_m1, *MCOLN1*), #4331182 (Hs00393705_m1, *SGPL1*), #4331182 (Hs01001189_m1, *SLC7A5*), #4331182 (Hs01027014_m1, *SPTLC2*), #4331182 (Hs00217867_m1, *SPTLC3*), #4331182 (Hs01105377_m1, *ST3GAL5*), #4331182 (Hs00383244_m1, *ZKSCAN3*), using the Light Cycler 480 II detection system (Roche Applied Science, Indianapolis, IN, USA). Expression values were normalised against three control genes *GAPDH*, *TBP* and *YWHAZ* of constant expression level under experimental conditions (2^−ΔΔ*C*t^ method). Determination of reference genes for real-time qRT-PCR was based on BestKeeper software analysis. While for all three implemented housekeeping genes, expression levels were comparable, data presented in Table 2 are fold-change normalised to *GAPDH*. For both DNA microarray and real-time qRT-PCR analyses, a fold change greater or equal to 1.3 and below and equal 0.7 was considered as a relevant criterion for genes being significantly differentially expressed, with a *p*-value of <0.05.

### 4.6. Measurement of Kinetics of GAG Synthesis

The kinetics of GAG synthesis was estimated by measurement of incorporation of glucosamine, D-[1-3H] (3H-GlcN) (Hartmann Analytic GmbH, Braunschweig, Germany) into GAG chains. HDFa and MPS cells were plated 2 × 10^4^ per well in a 48-well plate and incubated overnight to allow attachment. Cells were then preincubated for 48 h in standard DMEM supplemented with appropriate amounts of DMSO or in DMEM only (control cultures) and then labelled for 24 h with 10 μCi/mL of 3H-GlcN in DMEM without glucose and pyruvate (Thermo Fisher Scientific Inc., Paisley, UK) supplemented with 10% FBS and with or without DMSO.

After labelling, cells were washed six times with Dulbecco’s phosphate-buffered saline (DPBS, Thermo Fisher Scientific Inc., Paisley, UK) and digested for 3 h at 65 °C with 0.5% papain (Sigma-Aldrich Co. LLC., St. Louis, MO, USA) (prepared in 200 mM phosphate buffer (Na_2_HPO_4_–NaH_2_PO_4_), pH 6.4, containing 100 mM sodium acetate, 10 mM Na_2_EDTA and 5 mM l-cysteine). Incorporation of 3H-GlcN was measured in a MicroBeta^2^ scintillation counter (Perkin Elmer Inc., Waltham, MA, USA) and Quant-iT™ PicoGreen^®^ dsDNA reagent was used to determine DNA concentration in papain-digested samples. Incorporation of radioactive precursors was calculated per DNA amount (cpm/ng of DNA) and expressed as relative to control cultures (without treatment). To test the efficiency of DMSO on the kinetics of GAG synthesis, Kruskal–Wallis test was performed with Dunn–Bonferroni as a post hoc comparator with significance declared at *p* < 0.05 versus control cells without DMSO treatment.

### 4.7. Transmission Electron Microscopy (TEM)

In order to determine the effect of DMSO on cellular structures with special focus on the lysosomal system, 1.5 × 10^5^ HDFa and MPS III cells were plated in 12-well plates and allowed to attach overnight. Cells were then treated with DMSO at final concentrations 0.05%, 0.1%, 0.5%, 1%, 2% and 3% *v*/*v* and incubated for six days. Following PBS washing, cells were fixed with 2.5% glutaraldehyde, and then with 1% osmium tetroxide and 1% potassium hexacyanoferrate (III) followed by ethanol dehydration. Ultrasections of cells embedded in Epon 812 resin (Fluka Chemie GmbH, Buchs, Switzerland) were stained in lead citrate and uranyl acetate and examined under TEM (Philips CM100). All fibroblasts photographed using TEM program (Olympus Soft Imaging Solution, Münster, Germany) had intact membrane and visible cell structures (nucleus, mitochondria and lysosomes). At least 20 cells were examined and for each cell at least five random fields were assessed. Lamellar lysosomes and complex lysosomal structures (Figure 4) were counted by an individual blind to experimental conditions, per 100 μm^2^ cross-section of cell structures. Representative electron micrographs are shown at magnification 1650×.

## 5. Conclusions

As a result of our research, we managed to point out the role of dimethyl sulfoxide (DMSO) in gene expression modulation and glycosaminoglycan metabolism in lysosomal storage disorders on an example of mucopolysaccharidosis. In this report, we have tried to answer the question being asked and move forward to get further information and a better understanding of the problem.

## Figures and Tables

**Table 1:** Selected significantly overrepresented GO terms upon HDFa treatment with 0.05% DMSO. AmiGO analysis and Panther Classification System defined mostly enriched molecular functions, biological processes, cellular components and protein classes upon 0.05% DMSO HDFa treatment for 1, 24 and 48 h with false discovery rate (FDR) < 0.1, fold change (FC) ≤ 0.7 (blue columns) and ≥1.3 (red columns), *n* = 3 and *p* < 0.001. Each column represents numbers of DEGs involved in defined term and percentage gene hits against total number of genes (in brackets). Number of all overrepresented GO terms for each time point and class is presented in square brackets.

	Number of DEGs in Term					
GO Term	1 h		24 h		48 h	
***Molecular Function***	**[5]**	**[7]**	**[2]**	**[7]**	**[6]**	**[8]**
**binding** (GO:0005488)	10 (29.4%)	73 (31.1%)	4 (19.0%)	35 (22.0%)	27 (26.5%)	37 (27.0%)
**catalytic activity** (GO:0003824)	8 (23.5%)	53 (22.6%)	3 (14.3%)	44 (27.7%)	23 (22.5%)	35 (25.5%)
**transporter activity** (GO:0005215)	1 (2.9%)	9 (3.8%)		4 (2.5%)	8 (7.8%)	5 (3.6%)
**signal transducer activity** (GO:0004871)	1 (2.9%)	8 (3.4%)		2 (1.3%)	3 (2.9%)	4 (2.9%)
**receptor activity** (GO:0004872)	1 (2.9%)	12 (5.1%)		7 (4.4%)	2 (2.0%)	2 (1.5%
**structural molecule activity** (GO:0005198)	1 (2.9%)	7 (3.0%)		3 (1.9%)		3 (2.2%)
**translation regulator activity** (GO:0045182)		3 (1.3%)		2 (1.3%)		3 (2.2%)
***Biological Process***	**[9]**	**[13]**	**[9]**	**[12]**	**[11]**	**[11]**
**metabolic process** (GO:0008152)	15 (44.1%)	79 (33.6%)	6 (38.1%)	54 (34.0%)	33 (32.4%)	52 (38.0%)
**cellular process** (GO:0009987)	11 (32.4%)	113 (48.1%)	8 (28.6%)	70 (44.0%)	45 (44.1%)	52 (38.0%)
**cellular component organization or biogenesis** (GO:0071840)	2 (5.9%)	27 (11.5%)	2 (9.5%)	20 (12.6%)	5 (4.9%)	16 (11.7%)
**biological regulation** (GO:0065007)	3 (8.8%)	38 (16.2%)	4 (19.0%)	19 (11.9%)	10 (9.8%)	17 (12.4%)
**response to stimulus** (GO:0050896)	3 (8.8%)	32 (13.6%)	2 (9.5%)	16 (10.1%)	9 (8.8%)	15 (10.9%)
**developmental process** (GO:0032502)		27 (11.5%)	2 (9.5%)	16 (10.1%)	11 (10.8%)	8 (5.8%)
**localization** (GO:0051179)	2 (5.9%)	26 (11.1%)		10 (6.3%)	9 (8.8%)	13 (9.5%)
**multicellular organismal process** (GO:0032501)		19 (8.1%)		10 (6.3%)		4 (2.9%)
***Cellular Component***	**[4]**	**[7]**	**[3]**	**[7]**	**[5]**	**[7]**
**cell part** (GO:0044464)	8 (23.5%)	84 (35.7%)	4 (19.0%)	44 (27.7%)	29 (28.4%)	49 (35.8%)
**organelle** (GO:0043226)	6 (17.6%)	63 (26.8%)	4 (19.0%)	28 (17.6%)	21 (20.6%)	35 (25.5%)
**membrane** (GO:0016020)	1 (2.9%)	27 (11.5%)		11 (6.9%)	10 (9.8%)	16 (11.7%)
**macromolecular complex** (GO:0032991)	1 (2.9%)	30 (12.8%)	3 (14.3%)	12 (7.5%)	5 (4.9%)	19 (13.9%)
**extracellular region** (GO:0005576)		7 (3.0%)		7 (4.4%)	3 (2.9%)	3 (2.2%)
***Protein Class***	**[5]**	**[22]**	**[5]**	**[16]**	**[19]**	**[18]**
**transcription factor** (PC00218)	5 (14.7%)	24 (10.2%)	2 (9.5%)	8 (5.0%)	8 (7.8%)	7 (5.1%)
**nucleic acid binding** (PC00171)	4 (11.8%)	30 (12.8%)	2 (9.5%)	14 (8.8%)	7 (6.9%)	19 (13.9%)
**hydrolase** (PC00121)	3 (8.8%)	17 (7.2%)	1 (4.8%)	8 (5.0%)	5 (4.9%)	11 (8.0%)
**transferase** (PC00220)	2 (5.9%)	19 (8.1%)	1 (4.8%)	10 (6.3%)	5 (4.9%)	10 (7.3%)
**enzyme modulator** (PC00095)		10 (4.3%)		6 (3.8%)		7 (5.1%)
**receptor** (PC00197)		9 (3.8%)	1 (4.8%)	7 (4.4%)	3 (2.9%)	
**transporter** (PC00227)		7 (3.0%)		5 (3.1%)	7 (6.9%)	5 (3.6%)
**signaling molecule** (PC00207)		9 (3.8%)		7 (4.4%)	6 (5.9%)	7 (5.1%)
**transfer/carrier protein** (PC00219)	1 (2.9%)					

**Table 2:** Expression level of selected genes with the most changed activity profile after 1, 24 and 48 h treatment with 0.05% DMSO of HDFa. Normalised to untreated cells, relative genome microarray values ± standard deviation from *n* = 3 denote differences for samples incubated with 0.05% DMSO. Alterations in mRNA levels of genes are referred to FC ≤ 0.7 (**A**) and FC ≥ 1.3 (**B**) with the *p*-value < 0.05.

**(A)**					
**Time of Exposure to DMSO**					
**1 h**		**24 h**		**48 h**	
**Gene Expression Modulation**					
*BBS12*	0.58 ± 0.07	*ACVRL1*	0.60 ± 0.11	*ALG2*	0.60 ± 0.08
*CDKN1C*	0.60 ± 0.11	*APEG1*	0.63 ± 0.15	*ANKRD26*	0.64 ± 0.14
*DDIT4*	0.51 ± 0.10	*ATF2*	0.66 ± 0.02	*ANKRD38*	0.51 ± 0.15
*EBF1*	0.67 ± 0.05	*CCDC15*	0.68 ± 0.12	*ATP7B*	0.62 ± 0.17
*FLJ10246*	0.56 ± 0.08	*CHODL*	0.60 ± 0.14	*BAIAP2L1*	0.66 ± 0.12
*FLJ45244*	0.63 ± 0.11	*EDNRA*	0.61 ± 0.06	*BEX2*	0.59 ± 0.09
*GM2A*	0.61 ± 0.07	*FAM21A*	0.65 ± 0.12	*DAK*	0.59 ± 0.10
*HCFC2*	0.63 ± 0.06	*GGT7*	0.68 ± 0.05	*DHRSX*	0.61 ± 0.15
*HSPA1L*	0.57 ± 0.08	*LMNB1*	0.60 ± 0.04	*DKFZp761P0423*	0.61 ± 0.04
*KCNJ2*	0.62 ± 0.06	*MCPH1*	0.67 ± 0.03	*DNAJC1*	0.57 ± 0.08
*KIAA0100*	0.63 ± 0.18	*MFHAS1*	0.66 ± 0.00	*DNAJC3*	0.65 ± 0.02
*KRCC1*	0.66 ± 0.09	*PTHLH*	0.68 ± 0.07	*EIF2C4*	0.55 ± 0.04
*LIAS*	0.64 ± 0.02	*RNF141*	0.69 ± 0.02	*FAM127C*	0.66 ± 0.19
*LZTFL1*	0.67 ± 0.01	*SNORD16*	0.55 ± 0.02	*FAM83H*	0.52 ± 0.06
*MGA*	0.62 ± 0.11	*SSX2IP*	0.69 ± 0.00	*FKBPL*	0.59 ± 0.12
*NICN1*	0.63 ± 0.09	*SURF2*	0.67 ± 0.08	*FLJ11151*	0.62 ± 0.10
*PLAC9*	0.65 ± 0.02	*TAF13*	0.68 ± 0.13	*FLJ23834*	0.66 ± 0.04
*SLC40A1*	0.66 ± 0.02	*WIPF2*	0.65 ± 0.01	*FLJ35220*	0.64 ± 0.10
*SPIN1*	0.67 ± 0.03	*ZKSCAN3*	0.67 ± 0.01	*FLJ90086*	0.60 ± 0.02
*TAOK1*	0.67 ± 0.10	*ZNF417*	0.67 ± 0.10	*FRY*	0.61 ± 0.09
**(B)**					
**Time of Exposure to DMSO**					
**1 h**		**24 h**		**48 h**	
**Gene Expression Modulation**					
*ALG2*	1.64 ± 0.27	*BMP6*	1.65 ± 0.20	*ADAM9*	1.62 ± 0.33
*ANKS3*	1.62 ± 0.02	*FAM113B*	1.61 ± 0.21	*APCDD1L*	1.59 ± 0.47
*BET1L*	1.60 ± 0.23	*GFOD1*	1.73 ± 0.29	*BHLHB2*	1.56 ± 0.21
*CDC42EP3*	2.04 ± 0.04	*GSTM5*	1.55 ± 0.25	*CXCL1*	1.70 ± 0.03
*CDK8*	1.59 ± 0.19	*ITGBL1*	1.79 ± 0.53	*ERCC4*	1.54 ± 0.41
*DENND2C*	1.60 ± 0.15	*KCNJ16*	1.55 ± 0.35	*ETV3*	1.73 ± 0.41
*F5*	1.59 ± 0.01	*KIAA0746*	1.80 ± 0.08	*GFM2*	1.69 ± 0.06
*FLJ30058*	1.60 ± 0.27	*LENG8*	1.73 ± 0.19	*GNAQ*	1.59 ± 0.26
*GALNAC4S-6ST*	1.69 ± 0.45	*MEGF8*	1.72 ± 0.51	*HES4*	1.58 ± 0.33
*GRK6*	1.63 ± 0.37	*MESP1*	1.64 ± 0.38	*HPS3*	1.54 ± 0.14
*KLF10*	1.90 ± 0.11	*MT1G*	1.62 ± 0.27	*KLF10*	1.66 ± 0.32
*LMAN2*	1.70 ± 0.19	*MTE*	1.55 ± 0.46	*MAGEL2*	1.65 ± 0.45
*MAP6D1*	1.59 ± 0.45	*NAP1L3*	1.57 ± 0.32	*OXCT1*	1.65 ± 0.23
*MYBBP1A*	1.66 ± 0.48	*NDNL2*	1.66 ± 0.47	*PMS2*	1.80 ± 0.34
*NR4A1*	1.74 ± 0.25	*NEFM*	1.53 ± 0.37	*SNORD31*	1.78 ± 0.41
*SPIN2B*	1.62 ± 0.46	*SLC38A4*	1.69 ± 0.07	*SNX12*	1.73 ± 0.17
*TJP2*	1.84 ± 0.36	*STAC*	1.61 ± 0.13	*SPG21*	1.57 ± 0.45
*UBA2*	1.65 ± 0.20	*STK11*	1.55 ± 0.05	*STIM2*	1.58 ± 0.19
*UBA3*	1.60 ± 0.27	*ZBBX*	1.55 ± 0.02	*TBC1D23*	1.55 ± 0.43
*ZNF597*	1.62 ± 0.22	*ZSCAN16*	1.54 ± 0.21	*TMEM161A*	1.76 ± 0.24

**Table 3:** Expression patterns of GAG metabolism- and lysosome-associated genes in HDFa treated with 0.05% DMSO for 1, 24 and 48 h, identified in microarray and real-time qRT-PCR analyses (bolded are values of FC ≤ 0.7 and FC ≥ 1.3, *n* = 3, with the *p*-value < 0.05).

Gene Expression	Term	Genes	1 h		24 h		48 h	
			FC ± SD		FC ± SD		FC ± SD	
			Microarray	Real-Time qRT-PCR	Microarray	Real-Time qRT-PCR	Microarray	Real-Time qRT-PCR
**Upregulation**	Glycan biosynthesis and metabolism	*B4GALT1*	0.83 ± 0.0	0.81 ± 0.0	**1.32 ± 0.1**	**1.34 ± 0.3**		0.94 ± 0.0
		*GALNT2*	**1.46 ± 0.2**	**1.38 ± 0.1**	1.08 ± 0.1	1.12 ± 0.1		1.01 ± 0.4
		*GALNT4*	**1.50 ± 0.2**	**1.39 ± 0.1**	0.87 ± 0.1	0.93 ± 0.1	**1.34 ± 0.2**	1.05 ± 0.0
		*GALNT6*	1.11 ± 0.1	1.17 ± 0.1	0.91 ± 0.0	0.91 ± 0.0	**1.31 ± 0.1**	1.27 ± 0.1
		*GALNT12*	**1.33 ± 0.1**	1.29 ± 0.0	0.83 ± 0.0	0.91 ± 0.1		1.26 ± 0.0
		*MAN2C1*	**1.37 ± 0.0**	**1.42 ± 0.0**	0.90 ± 0.1	1.01 ± 0.1		0.78 ± 0.1
	N-Glycan biosynthesis	*ALG12*	1.12 ± 0.4	**1.30 ± 0.1**	**1.40 ± 0.0**	1.08 ± 0.2	0.78 ± 0.2	1.19 ± 0.1
	CS/DS biosynthesis	*CHST15*	**1.7 ± 0.4**	**1.73 ± 0.1**		1.14 ± 0.4	1.00 ± 0.2	0.98 ± 0.0
	HS synthesis	*EXT1*	**1.46 ± 0.1**	**1.48 ± 0.0**	0.94 ± 0.0	0.83 ± 0.1	0.88 ± 0.3	0.99 ± 0.0
	KS biosynthesis	*B4GALT1*	0.83 ± 0.0	0.81 ± 0.0	**1.32 ± 0.1**	**1.34 ± 0.0**		0.94 ± 0.0
		*CHST2*	**1.42 ± 0.1**	1.15 ± 0.5				
	GAG (CS, KS) degradation	*GALNS*	1.03 ± 0.2	0.98 ± 0.1	**1.37 ± 0.3**	**1.42 ± 0.2**	1.25 ± 0.1	1.02 ± 0.0
	SL metabolism	*SGPL1*		**1.34 ± 0.0**	**1.31 ± 0.3**	1.18 ± 0.3		0.88 ± 0.0
		*SPTLC2*	0.95 ± 0.0	0.91 ± 0.1		1.01 ± 0.0	**1.48 ± 0.4**	**1.31 ± 0.1**
		*SPTLC3*		1.17 ± 0.0		**1.31 ± 0.3**	**1.41 ± 0.2**	**1.37 ± 0.1**
	GSLs biosynthesis	*B4GALT1*	0.83 ± 0.0	0.81 ± 0.0	**1.32 ± 0.1**	**1.34 ± 0.0**		0.94 ± 0.0
		*ST3GAL5*	**1.32 ± 0.2**	**1.32 ± 0.0**	1.09 ± 0.1	1.03 ± 0.1	0.89 ± 0.0	0.97 ± 0.0
		*SLC7A5*	**1.32 ± 0.1**	**1.30 ± 0.0**		1.21 ± 0.5	1.07 ± 0.0	1.06 ± 0.0
	Sphingosine biosynthesis	*SPTLC2*	0.95 ± 0.0	0.91 ± 0.1	1.02 ± 0.2	1.01 ± 0.0	**1.48 ± 0.4**	**1.31 ± 0.1**
		*SPTLC3*		1.17 ± 0.0		**1.31 ± 0.3**	**1.41 ± 0.2**	**1.37 ± 0.1**
	Lysosome	*ARSK*		0.83 ± 0.2	1.11 ± 0.6	1.14 ± 0.0	**1.37 ± 0.1**	**1.44 ± 0.5**
**Downregulation**	N-Glycan biosynthesis	*ALG2*	1.64 ± 0.3	**1.61 ± 0.1**	0.97 ± 0.2	0.99 ± 0.0	**0.60 ± 0.1**	0.92 ± 0.0
	Lysosome	*GM2A*	**0.61 ± 0.1**	**0.58 ± 0.0**	0.98 ± 0.0	0.99 ± 0.0		**1.60 ± 0.1**
		*MCOLN1*	1.01 ± 0.2	0.99 ± 0.0	0.97 ± 0.0	0.99 ± 0.0	**0.68 ± 0.2**	0.81 ± 0.1
	Master repressor of autophagy	*ZKSCAN3*		**0.51 ± 0.0**	**0.67 ± 0.0**	0.92 ± 0.0	**0.69 ± 0.2**	0.88 ± 0.0
